# A Hybrid Clustering Algorithm for Identifying Cell Types from Single-Cell RNA-Seq Data

**DOI:** 10.3390/genes10020098

**Published:** 2019-01-29

**Authors:** Xiaoshu Zhu, Hong-Dong Li, Yunpei Xu, Lilu Guo, Fang-Xiang Wu, Guihua Duan, Jianxin Wang

**Affiliations:** 1 School of Computer Science and Engineering, Central South University, Changsha 410083, China; xszhu@csu.edu.cn (X.Z.); hongdong@csu.edu.cn (H.-D.L.); xu_yunpei@csu.edu.cn (Y.X.); jxwang@mail.csu.edu.cn (J.W.); 2 School of Computer Science and Engineering, Yulin Normal University, Yulin 537000, China; jgxygll@163.com; 3 Division of Biomedical Engineering and Department of Mechanical Engineering, University of Saskatchewan, Saskatoon, SK S7N5A9, Canada; faw341@mail.usask.ca

**Keywords:** single-cell RNA-seq, unsupervised learning, clustering, multikernel learning, *k* nearest neighbor, structure entropy

## Abstract

Single-cell RNA sequencing (scRNA-seq) has recently brought new insight into cell differentiation processes and functional variation in cell subtypes from homogeneous cell populations. A lack of prior knowledge makes unsupervised machine learning methods, such as clustering, suitable for analyzing scRNA-seq. However, there are several limitations to overcome, including high dimensionality, clustering result instability, and parameter adjustment complexity. In this study, we propose a method by combining structure entropy and *k* nearest neighbor to identify cell subpopulations in scRNA-seq data. In contrast to existing clustering methods for identifying cell subtypes, minimized structure entropy results in natural communities without specifying the number of clusters. To investigate the performance of our model, we applied it to eight scRNA-seq datasets and compared our method with three existing methods (nonnegative matrix factorization, single-cell interpretation via multikernel learning, and structural entropy minimization principle). The experimental results showed that our approach achieves, on average, better performance in these datasets compared to the benchmark methods.

## 1. Introduction

Gene expression profiles can represent the development stage of cells and the differentiation state of cells. For example, based on gene expression profiles, the classification of colorectal cancer can find subtypes to display resistance to therapy [1,2,3]. Gene expression across tissues has been described, which can be used to build complex networks and understand the heterogeneity of human tissues [4,5,6]. Traditional gene expression of bulk cells is obtained by sequencing a large number of cells that are commonly a mixture of different cell types or tissues [7,8]. Single-cell RNA sequencing (scRNA-seq) [9,10,11] is able to address the limitation of conventional bulk sequencing approaches. For example, bulk sequencing technology measures the mean gene expression of multiple cells and discards the difference of cells [12,13]. Single-cell RNA sequencing has attracted a great amount of attention for the following characteristics: (1) It can sequence more samples than traditional bulk methods and obtain more raw material for downstream analysis [14]; (2) it can be clearly observed that scRNA-seq data is sparse. The average sparsity may reach 50% [15]. The number of samples is usually from tens to hundreds of thousands, which is relatively smaller than other types of datasets, such as image datasets. The gene dimension of scRNA-seq data is usually tens of thousands, which is relatively very high. Meanwhile, the high dimension of datasets makes it difficult to measure the difference of gene expression patterns between cell types; (3) it has greater capability to explore cell type differentiation, resulting in rare cell populations and new cell subtypes. Based on the characteristics mentioned above, scRNA-seq data can be used to study embryonic development, population lineages and cancer treatment [16,17,18,19,20]. Thus, to analyze scRNA-seq data, we would face the following computational challenges: Sparsity, small sample size, high dimensionality, and lack of prior knowledge.

Clustering is a very effective method to analyze scRNA-seq data, which commonly includes two types of methods according to whether prior knowledge is needed or not [21,22,23]. Some existing methods train the labeled scRNA-seq data and tested data to predict cell types, in which prior knowledge is needed. For example, Shekhar et al. [24] identified cell types based on retinal bipolar neuronal scRNA-seq data from 455 mice. They used principal component analysis to reduce dimension; the *k* nearest neighbor [25,26] and Louvain community detection method [27,28,29] were used to identify cell types. It is well known that the *k* nearest neighbor algorithm is a classical classification method, in which the *k* nearest neighbors of a node are selected by computation the distance between the node and the *k* neighbors. Louvain community detection is a well-established graph algorithm, which can find the community modules from complex networks using the greedy optimization method and modularity maximization. Another type of method clusters the unlabeled scRNA-seq data to predict cell types without prior knowledge about cell types. One commonly used method is hierarchical clustering (Llorens et al. [30], Darmains et al. [31]). Llorens et al. found the principles underlying quiescent neural stem cells and lineage priming. They identified a dormant neural stem cells subpopulation, in which distinct combinations of lineage-specific transcription factors were expressed. Darmains et al. calculated the similarity matrix using the Pearson correlation coefficient to generate minimum spanning tree, constructed a cell network through random walk methods, and identified the subgroup by hierarchical clustering [32]. They identified gene sets that were significantly differentially expressed between fetal and adult neurons. The gene sets showed a difference expression gradient, reflecting the transition state between replicating and quiescent fetal neuronal populations. Another commonly used method is K-means (Shin et al. [33]). Shin et al. improved Waterfall, which was a pipeline that used K-means [34,35] clustering to build a trajectory and assign an individual cell a pseudotime based on each cell’s proximity to the cluster-derived trajectory. By researching the subgranular zone, they determined the trajectory. In addition to these methods, Xu et al. [36] automatically calculated the cluster number and effectively clustered cell types using the shared nearest neighbor to measure similarity and construct graph, and the quasi-clique-based algorithm to determine the number of clusters and find a clique, named SNN-clique, which can identify different dense clusters. The clustering results reflected the cell types or origins with high accuracy. Shao et al. [37] used nonnegative matrix factorization in a cell-centered direction to cluster cell subtypes based on three mouse scRNA-seq datasets. Nonnegative matrix factorization can decompose the gene expression matrix into two nonnegative matrices: The basis matrix (contribute to find sample clusters) and the coefficient matrix (contribute to find feature genes), to find natural subgroups. They used sparseness and entropy to determine the rank and the meaningful number of subpopulations. Without prior dimension reduction, they revealed the signature genes about cell subtypes. Kiselev et al. [38] constructed a consensus matrix using the cluster-based similarity partitioning algorithm and clustered six public gold standard scRNA-seq datasets and six silver standard scRNA-seq datasets. They calculated the Euclidean, Pearson, and Spearman metrics between the cell pairs to construct distance matrices, whose dimension was reduced using either principal component analysis or the eigenvectors of the associated graph Laplacian matrix. Wang et al. [39] proposed a novel similarity measurement method, single-cell interpretation via multikernel learning, using kernel function and spectral clustering, which achieved a high clustering performance. In the previous approaches, a lot of efforts have been focused on obtaining robust and significant clustering results, and complex similarity measurement methods or clustering algorithms were designed. Specially, some methods represented instability in different datasets and obviously depended on adjusting parameters. 

To address the aforementioned issue in unsupervised learning methods based on scRNA-seq datasets, we explored an effective and robust clustering method in this study using graph theory and structure entropy theory. Our proposed method included three steps: Firstly, the similarity matrix of cell samples was constructed by learning different weights for multiple kernels to measure cell-to-cell distances. Secondly, the weighted cell network was constructed with the *k* nearest neighbor algorithm; the weight of edges was determined by the similarity matrix. Thirdly, clustering was performed using the two-dimensional structure entropy minimum principle. On eight public scRNA-seq datasets, the performance of the presented method was investigated in terms of two evaluation metrics: Normalized mutual information and adjusted rand index. From the experiment results, we found that our approach achieved the best average performance in these datasets compared to other methods.

## 2. Materials and Methods 

**Figure 1:**
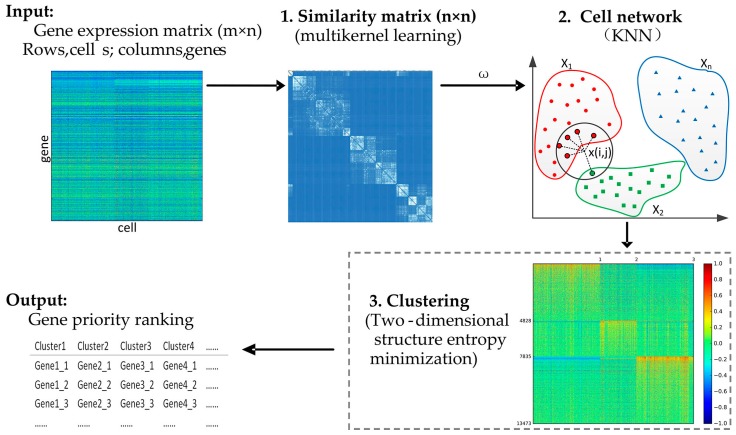
The mechanism of the SSE (single-cell structure entropy minimization principle) algorithm. The input is a gene expression matrix. The SSE algorithm includes three steps: (**1**) The similarity is calculated by multikernel learning; (**2**) the cell network is constructed by KNN (*k* nearest neighbor); (**3**) clustering is implemented using the structure entropy minimized principle. Lastly, gene priority ranking results as an output.

A framework of our proposed method (single-cell structure entropy minimization principle, SSE) is presented in Figure 1. This is a hybrid clustering algorithm based on multikernel learning, *k* nearest neighbor (KNN), and structure entropy. It is well known that there are various methods to cluster high dimensional data into interpretable subparts, among which we applied and combined two novel methods, multikernel learning and structure entropy, and KNN. Firstly, single-cell interpretation via multikernel learning (SIMLR) is a novel similarity measurement method, which is insensitive to the parameter pairs (*k*,σ) and the number of kernels. Moreover, we tested our method with different values of parameter *k* (*k* = 5, 10, 15, 20, 25, the default value is 10) based on two datasets with a typically accurate label and found that our algorithm was also insensitive to the value of parameter *k*. Multikernel learning can best fit the data structure and enforce block structures in similarity calculation by integrating multiple kernels [39,40]. Secondly, KNN is a classical and very popular method in clustering for its easy-to-understand implementation and significant classification performance [41,42], and it has been voted as one of the top ten data mining algorithms. KNN can represent the sample network by constructing a KNN graph and detect the community quickly [43,44,45,46]. The KNN method has only one parameter *k* to adjust. Thirdly, entropy can be used to measure the complexity of networks and represent the stability of a system in which the lower the entropy, the more stable the system is. Thus, the principle of structure entropy minimization can detect the natural communities in networks [47,48]. In this study, we tried to use their advantages to do the research on identification of cell types and SSE inherits three main advantages over these compared methods. First, it does not need to decide the parameter *k* in the KNN algorithm by combining multikernel similarity learning. Second, SSE can apply to cluster scRNA-seq data without prior knowledge about the true number of clusters. Third, SSE does not need to adjust model parameters using the default values of parameters from SIMLR.

### 2.1. Cell-to-Cell Similarity Measurement

Cell-to-cell similarity measurement plays an important role in cell sample clustering. The common similarity calculation methods are as follows: Euclidean distance, Spearman correlation, Pearson correlation coefficient, Jaccard similarity, Minkowski distance, and so on. Beyond that, some researchers proposed novel methods for distance or similarity calculation, such as Kiselev et al. [38] and Wang et al. [39]. We calculated the cell-to-cell similarity by kernel-based learning method, proposed by Wang et al. [39], which would overcome the problem that some distance calculation methods were affected by data distribution, such as Minkowski distance. We chose this similarity measurement method mainly for the following reasons: First, SIMLR was recently referenced and considered as an efficient similarity measurement method [49,50,51]. Second, SIMLR had the following main advantages for similarity measurement: (1) It provided a distance metric by combining multiple kernels; (2) it employed a rank constraint to address the dropout events, in which it enforced a block structure and obtained a more accurate similarity matrix for downstream steps; (3) the parameters of SIMLR were (*k*,σ) and the number of kernels, and the empirically results showed that it was insensitive to the parameters. 

Here, given a gene expression matrix as an input, rows correspond to cells, while columns correspond to genes. Multikernel learning was used to calculate the distance between the cells and construct a similarity matrix in the following two steps [39]: 

(1) To compute the distance between a pair of cells, the distance formula was detailed in the literature, in which each weight value described the importance of each kernel. Gaussian kernels were used here, and each kernel was decided by a parameter pair (*k*,σ). The experiments showed that the method was insensitive to the parameter pair. The parameter pair was set to default values.

(2) To construct a similarity matrix based on an optimization framework over *S*, *L*, and *w*, where *S* is a similarity matrix, *L* is an *N×C* rank-enforcing matrix, and *w* is the weight of kernels, the optimization algorithm was detailed in the literature.

### 2.2. Cell Network Construction

KNN is a popular method for its significant ability to present network structure and simple implementation. Here, we used the popular KNN algorithm [52]. Because the result matrix of multikernel similarity learning was a sparse matrix, which had reserved the nodes with larger similarity, we did not need to test a special value of *k*, and kept all the edges to construct a graph. We constructed a weighted undirected cell network *G* = (*V*, *E*). Suppose that *c*_1_, *c*_2_, …, *c_n_* were *n* cells, and *g*_1_, *g*_2_, …, *g_m_* were *m* genes. We denoted the input gene expression matrix *X* = [*x_ij_*], with rows representing cells and columns representing genes. Thus, its *i*th row and *j*th column were denoted as *c_i_* and *g_j_*, respectively. 

The algorithm for constructing cell network is as follows:

(1) For each *i* from 1 to *n*, a vector (*x*(*i*, 1), *x*(*i*, 2), …, *x*(*i*, *m*)) represented the genes expression of cell *c_i,_* and the gene number *j* is from 1 to *m*. The sample *x*(*i*, :) was one node of network G.

(2) Distance between *x*(*i*, :) and *x*(*i*’, :) was calculated, denoted *w* (*i*, *i*’), which was the weight of edge between *x*(*i*, :) and *x*(*i*’, :).

(3) For each *i* from 1 to *n*, all edges adjacent to the *x*(*i*, :) were reserved.

In the traditional KNN method, the choice of the value of *k* is a challenge. Wang et al. chose *k* = 3 based on experimental experience. Li et al. [53] used the one-dimension structure entropy minimization principle to determine the value of *k*, but this method would not sometimes find *k* in a few scRNA-seq data. In our method, the value of *k* would not be specified through testing an empirical value from the above analysis. The details were described later in the article.

The pseudocode for the used Algorithm 1 is as follows:

**Algorithm 1** Hybrid clustering algorithm pseudocode**Input:** Gene expression matrix *X* = [*x_ij_*], row is cells, column is genes;
**Process:**
1:*n* = the number of cells, *m* = the number of genes; 2:**for***i* = 1, 2, … *n*3:  **for**
*i*’ = 1, 2, … *n*4:    calculate distance between *x*(*i*,:) and *x*(*i*’,:) using SIMLR algorithm;5:here, we get similar matrix for each cells, denoted *w*(*i*,*i*’);6:**for***s* = 1, 2, … *n*7:  reserve all values in *w*(*s*,:), and set 0 for other values;8:here, we get sparse matrix, denoted S(*i*,*i*’);9:**Output:** S(*i*,*i*’) will be used to construct graph in SSE algorithm 

### 2.3. Cell Types Identification 

Entropy can be used as a metric for representing object uncertainty, as well as the information needed to determine the event. The smaller the entropy is, the more orderly the system is. According to Shannon’s entropy function, entropy is defined as follows:

(1) $$H\left(p_{1},\cdots ,p_{n}\right)=-\sum_{i=1}^{n}p_{i}\log_{2}p_{i}$$

where *p_i_* is a probability that event *i* occurs with $\sum p_{i}=1$. $-\log_{2}p_{i}$ bits needed to represent a variable that can take one of 1/*p_i_* values if 1/*p_i_* is a power of 2.

In a cell network, communities can be detected when entropy is minimized. However, entropy does not have enough information to measure the complexity of a network, so additional information needs to be added. In order to address this issue, we employed the structure entropy minimization principle proposed by Li et al. [53]. The principle of graph structure entropy and the criteria used for partitioning the overall network into cell subpopulations are described as following. The detail of structure entropy definition and minimization can be found in [53].

The graph structure entropy can provide a matrix of the dynamical complexity of the network. For a graph *G*, the *k*-dimensional structural entropy is defined as the fewest bits needed to describe the *k*-dimensional space information of the node, which is obtained from random walk in *G*. To detect the natural communities, two-dimensional graph structural entropy is defined as the average number of bits required to determine the code (*i*,*j*) of the node. 

Suppose that ℤ = {*X*_1_, *X*_2_, ⋯, *X_L_*} was a sub region of node set V, and each of *X*_1_, *X*_2_, ⋯, *X_n_* was defined as a community in graph *G.* Then, *X* (*i, j*) encoded node *v*, in which *i* was the code of *v* in local community *X_n_*, and *j* was the code of community *X_n_* in global *V*. From the abovementioned, the structure entropy was defined as Equation (2):

(2) $$H^{P}(G)=-\sum_{l=1}^{L}\frac{Vol_{l}}{2e}\sum_{i=1}^{n_{l}}\frac{d_{i}^{l}}{Vol_{l}}\log_{2}\frac{d_{i}^{l}}{Vol_{l}}-\sum_{l=1}^{L}\frac{e_{j}}{2e}\log_{2}\frac{Vol_{l}}{2e}$$

where *L* was the number of community *X_l_* in ℤ, *n_l_* was the number of node in community *X_l_*, *d_i_^l^* was the degree of the *i*-th node of *X_l_*, *Vol_l_* was the sum of the degrees of the nodes in community *X_l_*, and *e_j_* was the number of edges with just one endpoint in community *X_l_*. The structure entropy of graph *G* was defined as Equation (3), and minimizing the structure entropy of the graph would achieve the natural community structure of the network:

(3) $$H(G)=\underset{P}{\min}\{H^{P}(G)\}$$

where ℤ run over the subregion of *G*.

### 2.4. Feature Gene Selection

In the gene expression matrix, each gene is an attribute of a cell. The gene expression value contributes to cluster cells and affects the result significantly due to its high dimensionality. Some methods implemented dimension reduction, which is gene feature extraction, to get better clustering results. Nevertheless, bias would be introduced and relevant genes may be dropped. The technique and biological noise would lead to a poor result, such as only the first few components of principal component analysis (PCA) not being able to distinguish the subpopulation unambiguously [54,55]. Our approach differed from those methods, whereas the feature genes were selected to get the marker genes after clustering. We computed the average of certain gene expression values in every community to determine which community a gene belongs to. Then, genes in a community were sorted in descending order by the gene expression value. The top k genes were selected to be the marker genes relevant to subpopulation. 

### 2.5. Time Complexity Analysis

The most time-consuming step of SSE is to cluster using two-dimension structure entropy minimization, which requires *O* (*n*^2^) time. Here, *n* is the number of cells. Since the number of cells is usually far less than the number of genes, this step is still fast. In addition, the time complexity to construct a cell network is *O* (*n*) using a KNN graph. For optimization framework solutions for *S*, *L* and *w* iteratively in the similarity measurement step, the time complexity is *O* (*Tkn*), where *T* is the number of iterations and *k* is the number of neighbors. 

### 2.6. Datasets Description

Single-cell RNA-seq data based on cell type differentiation are crucial for understanding cell linage relationships and predicting the relationship between diseases and treatments. Thus, we executed SSE on eight test datasets, which are summarized in Table 1. These datasets were downloaded from EMBL-EBI (https://www.ebi.ac.uk/) or the NCBI Gene Expression Omnibus (GEO) repository (https://www.ncbi.nlm.nih.gov/geo/), among others. 

## 3. Experiments and Results 

To demonstrate the performance of the proposed method SSE, we carefully compared it with three unsupervised learning methods for scRNA-seq data analysis: Nonnegative matrix factorization (NMF), SIMLR, and structural entropy (SE) minimization principle. All these algorithms were run on Windows 7. To perform SSE, we used the R code to implement a similarity matrix by multikernel learning algorithms, which are given in detail in [39]. We also used a JAVA code to implement structural entropy minimization principle algorithms, which are given in detail in [53]. The heat maps were drawn by a matplotlib package in Python, version 2.7.12 [64].

### 3.1. Performance Evaluation 

To make the comparison fairly, we ran all methods with the commonly used eight datasets which were analyzed in other methods. In the same way, we compared these methods based on two evaluation metrics: Normalized mutual information (NMI) and adjusted Rand index (ARI). The true number of populations, abbreviated as ‘gold standard’ cluster numbers, was applied on computing the NMI value and ARI value. The number of categories of datasets was selected on the basis that one could be highly confident in the cell-labels, as they represent cells from different conditions or lines, and thus we considered them ‘gold standard’. The ‘gold standard’ cluster number of each testing dataset is shown in Table 1. 

NMI [65] is commonly used to evaluate the consistency between the obtained cluster results and the true labels of the cells. NMI is defined as follows:

(4) $$NMI(X,Y)=2\frac{I\left(X;Y\right)}{H\left(X\right)+H\left(Y\right)}$$

(5) $$I\left(X;Y\right)=\sum_{y\in Y}\sum_{x\in X}p\left(x,y\right)\log\left(\frac{p\left(x,y\right)}{p\left(x\right)p\left(y\right)}\right)$$

(6) $$H\left(X\right)=-\sum_{i=1}^{n}p\left(x_{i}\right)\log_{b}p\left(x_{i}\right)$$

where *I* (*X; Y*) is the mutual information between clustering *X* and *Y*, and *H*(*X*) is the entropy of the clustering *X*. *p*(*x, y*) is the joint probability distribution function of *x* and *y*. *p*(*x_i_*) is the probability distribution function of *x_i_*.

ARI [37] is commonly used to evaluate the agreement between the predicted clusters and the true categories. ARI is defined as follows:

(7) $$ARI=\frac{\sum_{ij}\left(\begin{array}{c} n_{ij}\\ 2 \end{array}\right)-\left[\sum_{i}\left(\begin{array}{c} a_{i}\\ 2 \end{array}\right)\sum_{j}\left(\begin{array}{c} b_{i}\\ 2 \end{array}\right)\right]/\left(\begin{array}{c} n\\ 2 \end{array}\right)}{\frac{1}{2}\left[\sum_{i}\left(\begin{array}{c} a_{i}\\ 2 \end{array}\right)+\sum_{j}\left(\begin{array}{c} b_{i}\\ 2 \end{array}\right)\right]-\left[\sum_{i}\left(\begin{array}{c} a_{i}\\ 2 \end{array}\right)\sum_{j}\left(\begin{array}{c} b_{i}\\ 2 \end{array}\right)\right]/\left(\begin{array}{c} n\\ 2 \end{array}\right)}$$

where *a*, *b*, *c*, and *d* are calculated as follows, respectively.

*a*: A number of pairs of objects are placed in the same group in *X* and in the same group in *Y*;

*b*: A number of pairs of objects are placed in the same group in *X* and in a different group in *Y*;

*c*: A number of pairs of objects are placed in the same group in *Y* and in a different group in *X*; 

*d*: A number of pairs of objects are placed in a different group in *X* and in a different group in *Y*;

*n*: The number of the elements (cells).

The overlap between *X* and *Y* can be formed in a contingency table, and *n_ij_* are the values from abovementioned contingency table; *a_i_* is the *i*-th row of the contingency table and *b_j_* is the *j*-th column of the contingency table.

We compared the performance of our method SSE to NMF, SIMLR, and the structural entropy minimization principle (SE) in terms of NMI and ARI. The results of NMI are listed in Table 2, while the results of ARI are listed in Table 3. It is worth mentioning that all of these methods were performed with default parameters, without any parameter optimization. The parameter pair (*k*, σ) of SIMLR was set to default values. SE also had parameter σ’ (different from that of SIMLR), with σ’ defaulting to 1/2n; the number of clusters calculated by SE, denoted as *k*’, depends on σ’ by one dimensional structure entropy minimization. For SE, when *k*’ could not be easily determined at the default value of σ’, different σ’ values in {1/n, 2/3n, 1/2n, 2/5n, 1/3n} were tested to determine *k*’.

From Table 2; Table 3, we can see that a specific method for domain-specific scRNA-seq dataset performed well. SSE had the best average performance and achieved a better performance for some datasets, such as the Biase, Deng, Pollen, Patel, and Chung datasets. SE performed better for the Yan and Treutlein dataset. SIMLR achieved a better performance for the Deng and Pollen datasets. NMF performed better for the Ramskold dataset. 

Taken together, the above results indicated that SSE was a robust method with the best average performance, which would be applied for clustering analysis to identify cell types. Especially, these results provided evidence that SSE was a simple and promising tool for clustering analysis, which did not need to adjust complex parameters, including the value of *k*. Meanwhile, we used the Mann–Whitney U test, which is a commonly used nonparametric test method, to test whether our method significantly outperformed others. The results showed that the improvement is insignificant. However, it should be noted the improvement varies a lot for different datasets. For example, our method achieves much better results on the Biase data, but the improvement is less significant on the Chung data; on the Treutlein data, our method performed worse than others.

To describe the overlap and relationship of the four methods, the cluster results comparison between SSE and NMF, SIMLR, and SE in terms of NMI and ARI were calculated, and the results are shown in Supplementary Tables S1 and S2.

### 3.2. Cluster Result Analysis

**Figure 2:**
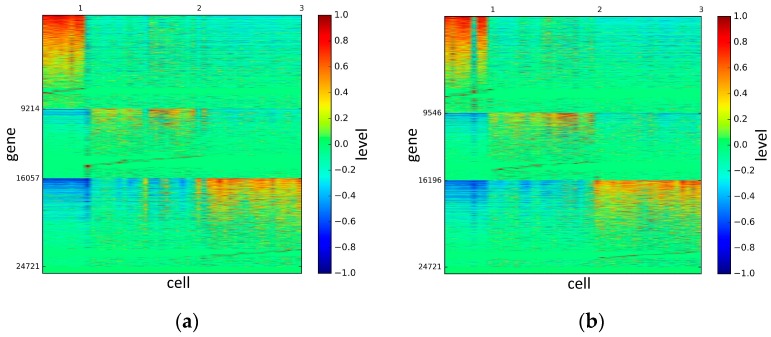
The heat maps of Biase datasets.

**Figure 3:**
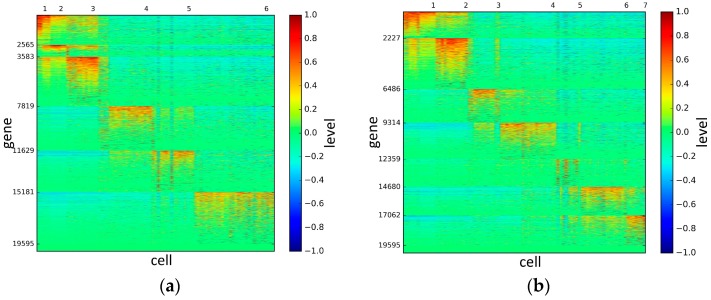
The heat maps of Yan datasets.

**Figure 4:**
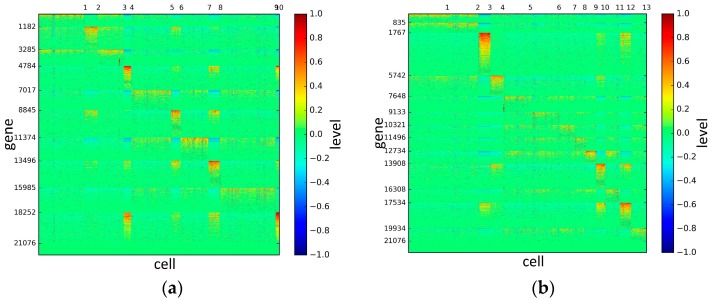
The heat maps of Deng datasets.

**Figure 5:**
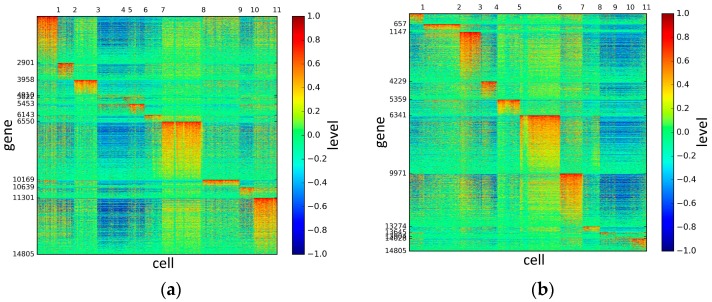
The heat maps of Pollen datasets.

**Figure 6:**
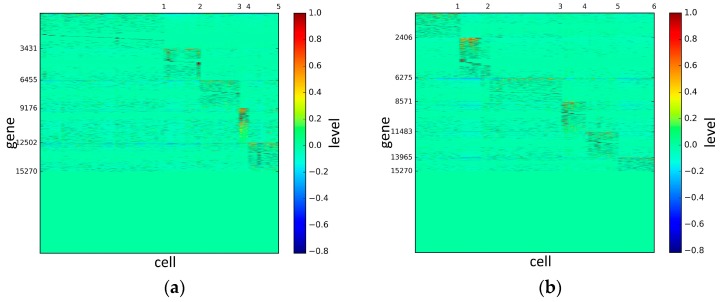
The heat maps of Treutlen datasets.

**Figure 7:**
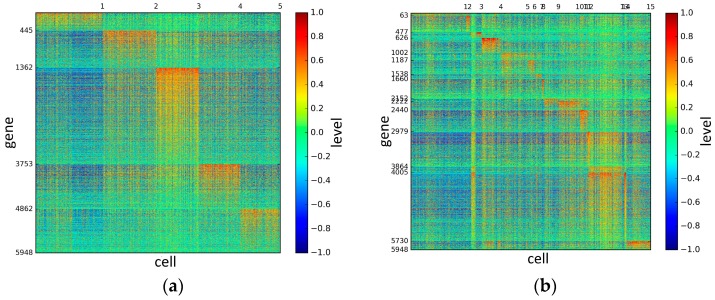
The heat maps of Patel datasets.

**Figure 8:**
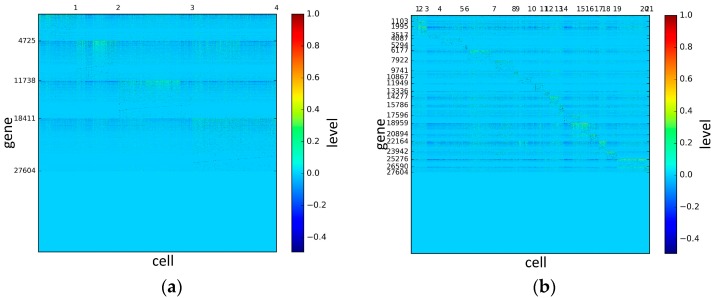
The heat maps of Chung datasets.

**Figure 9:**
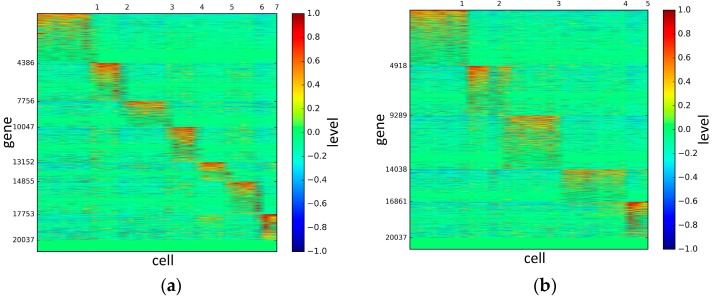
The heat maps of Ramskold datasets.

To represent and analyze the cluster results, the true types and cluster heat maps of the eight datasets were provided, giving the visualization of how these cell samples are clustered, as shown in Figure 2, Figure 3, Figure 4, Figure 5, Figure 6, Figure 7, Figure 8 and Figure 9, in which (a) is the heat map of true types with labels and (b) is the heat map of cluster result using SSE method. The x-coordinate represents the cell samples, the y-coordinate represents the gene expression values, and the top horizontal line marks the number of categories.

According to the heat maps, we found that our method could cluster the samples unambiguously. The cluster numbers were marked above the top horizontal line. Clear blocks appear in the diagrams. Each of the blocks was the high expression gene set in one cluster, that is, a feature gene set. Moreover, we observed that SSE achieved different cluster numbers than the other competing methods. The detail of cluster number results is shown in Table 4. Especially in the Patel dataset and Chung dataset, this phenomenon was more obvious. For the Patel dataset, the gold standard number was 5, while it was 15 in the SSE result. Meanwhile, compared to other methods, SSE achieved the best NMI value of 0.599. For the Chung dataset, the gold standard number was 4, while it was 21 in the SSE result. Meanwhile, compared to other methods, SSE achieved the best NMI value of 0.334. 

**Figure 10:**
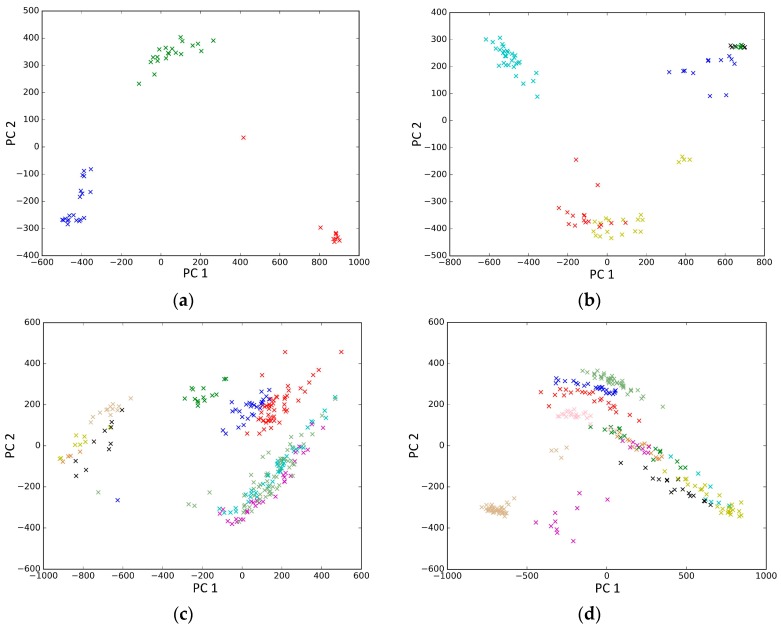

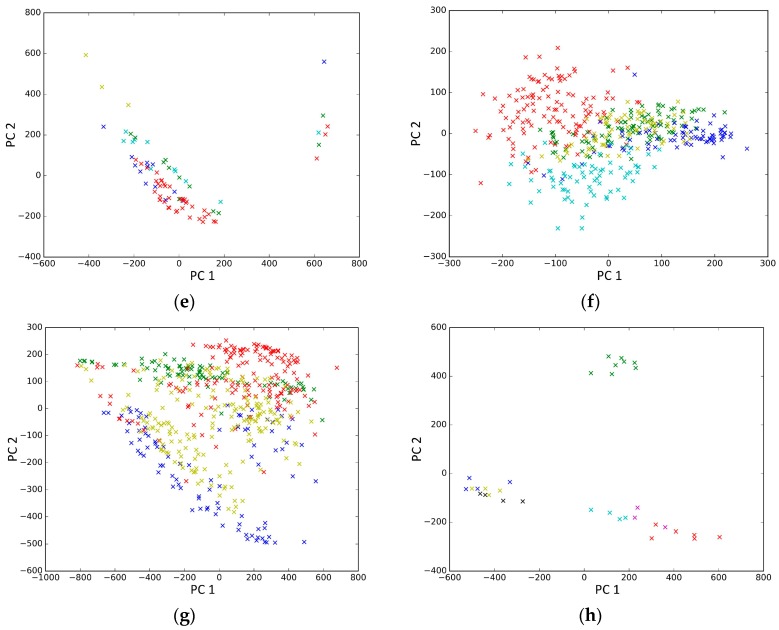
The scatter diagram of eight datasets by principal component analysis (PCA). (**a**) Biase; (b) Yan; (**c**) Deng; (**d**) Pollen; (**e**) Treutlein; (**f**) Patel; (**g**) Chung; (**h**) Ramskold.

PCA is a popular tool to identify the subgroups from scRNA-seq data, of which the first two components are commonly performed for visualization [58]. The first two components capture the highest percentage of variance, which means greater information, so we used them to visualize the eight datasets after binary log-transformation and centering of the scRNA-seq data. The scatter diagram of eight datasets by PCA is shown in Figure 10. In the experiments, each sample point in the same category was assigned the same color according to its true label. From Figure 10, some remarkable phenomena can be observed: (1) Limited to the difference of inherent attributes in each dataset, the performance of PCA method varied greatly over different datasets. Note that the Biase dataset was clustered clearly into three groups, which was in accordance with the true clusters. However, it was unfortunate that the PCA method did not work well in other datasets with higher heterogeneity; (2) SSE had an excellent clustering performance both in the Biase and Pollen dataset, i.e., several block structures were revealed in the gene map, which indicated that SSE better discovered the true clusters. We can observe that there were more blocks in the other five datasets from the gene maps; this phenomenon can particularly be observed in the Patel and Chung datasets. Because there was no cluster number as input as in NMF and SIMLR, SSE and SE found more or less clusters based on scRNA-seq data; this aspect deserves further investigation; (3) the marker genes in each cluster could be specified explicitly via the SSE method, but the PCA method could not get it. Finally, we observed that some datasets were clearly separated, such as the Biase dataset, and most datasets were indistinguishable.

Moreover, to describe the results of dimensionality reduction more fully, we applied another nonlinear dimensionality reduction method, t-SNE (t-distributed stochastic neighbor embedding). The scatter diagram of eight datasets by t-SNE can be found in Supplementary Figure S1. To better spot out possible clustering, we also presented the visualization of single cells in 3D space using the first three principal components (Supplementary Figure S2).

## 4. Discussion

Single cell RNA-seq data posed a challenge to cluster approaches for exploring new cell subtypes and rare cell populations without prior knowledge. Scialdone et al. clustered mouse embryonic stem cells, suffering from the limitation of the dependence on known data as training dataset. As a matter of fact, most datasets were lacking prior knowledge. In addition, as similarity calculation plays an important role in clustering results, complex similarity measurement algorithms were designed to get high accurate clusters. Here, we explored graph theory and the structure entropy minimization principle for the purpose of subgroup identification in scRNA-seq data. Instead of using conventional hierarchical clustering, here we focused on minimizing the structure entropy to find the natural communities in cell networks. We found that SSE correctly clustered cells to biologically meaningful subgroups. Compared to NMF, SIMLR, and SE, SSE could produce the cluster results as stable communities that were straightforward to interpret. Remarkably, SSE performed well even without prior dimension reduction, such as extraction feature genes using PCA.

As can be seen from our analysis, in the SSE method, we constructed cell networks using KNN, as Xu et al. did. However, Xu et al. had to adjust a set of parameters *k*, *r*, and *m* to improve cluster performance. Nevertheless, SSE only had the parameter pair (*k*,σ) of SIMLR with default values. Beyond that, there were no other parameters to be adjusted in the steps of network construction and clustering.

In addition, SSE proved very robust when it was applied to scRNA-seq datasets. By analyzing eight datasets, we found that SSE showed the best average performance in terms of NMI and ARI compared to the three competing approaches. In conclusion, our study showed that SSE was an effective and robust clustering method for scRAN-seq dataset.

## Figures and Tables

**Table 1:** List of datasets and their attributes.

GSE/ID	Datasets	Tissue	Number of Cells	Number of Genes	Amount of Population	References
GSE57249	Biase	Mouse embryo cell	49	25,384	3	Biase et al., 2014 [56]
GSE36552	Yan	Human embryo cell	90	20,214	6	Yan et al., 2013 [57]
GSE45719	Deng	Mouse embryo cell	259	22,147	10	Deng et al., 2014 [58]
E-MTAB-2805	Pollen	Human different tissues (stem cell)	249	14,805	11	Pollen et al., 2014 [59]
GSE52583	Treutlein	Mouse lung epithelial cell	80	23,129	5	Treutlein et al., 2014 [60]
GSE57872	Patel	Human glioblastoma cells	430	5948	5	Patel et al., 2014 [61]
GSE75688	Chung	Human breast cancer and lymph node metastasis cells	518	41,821	4	Chung et al., 2017 [62]
GSE38495	Ramskold	Human cancer cell	33	21,042	7	Ramsköld et al., 2012 [63]

**Table 2:** Cluster performance comparison of NMF (nonnegative matrix factorization), SIMLR (single-cell interpretation via multikernel learning), SE (structural entropy minimization principle), and SSE (single-cell structural entropy minimization principle) in terms of NMI (Normalized mutual information).

Datasets	NMF	SIMLR	SE	SSE
Biase	0.322	0.673	0.554	**0.721**
Yan	0. 673	0.727	**0.776**	0.747
Deng	0.509	**0.676**	0.635	**0.676**
Pollen	0.944	**0.950**	0.781	**0.950**
Treutlein	0.277	0.276	**0.344**	0.270
Patel	NA	0.576	NA	**0.599**
Chung	0.196	0.283	0.322	**0.334**
Ramskold	**0.831**	0.818	0.596	0.772
Average	0.536	0.622	0.573	**0.634**

**Table 3:** Cluster performance comparison of NMF, SIMLR, SE and SSE in terms of ARI (Adjusted Rand index).

Datasets	NMF	SIMLR	SE	SSE
Biase	0.244	0.682	0.682	**0.742**
Yan	0.519	0.487	0.477	**0.524**
Deng	0.312	0.364	0.388	**0.386**
Pollen	0.981	**0.943**	0.613	**0.943**
Treutlein	0.262	**0.229**	0.183	0.155
Patel	NA	0.527	NA	**0.553**
Chung	0.134	0.136	**0.200**	0.158
Ramskold	**0.686**	0.683	0.344	0.613
Average	0.448	0.506	0.412	**0.509**

**Table 4:** The number of clusters in the ‘gold standard’ and four methods.

Datasets	Gold Standard	NMF	SIMLR	SE	SSE
Biase	3	3	3	5	3
Yan	6	6	6	11	7
Deng	10	10	10	8	13
Pollen	11	11	11	7	11
Treutlein	5	5	5	4	6
Patel	5	5	5	NA	15
Chung	4	4	4	11	21
Ramskold	7	7	7	3	5

## Supplementary Materials

The following are available online at http://www.mdpi.com/2073-4425/10/2/98/s1, Figure S1: The scatter diagram of eight datasets by t-SNE, Figure S2: The three-dimensional scatter diagram of eight datasets by PCA, Table S1: Cluster results comparison between SSE and NMF, SIMLR, SE in terms of NMI, Table S2: Cluster results comparison between SSE and NMF, SIMLR, SE in terms of ARI.

## References

[1] Calon A., Lonardo E., Berenguer-Llergo A., Espinet E., Hernando-Momblona X., Iglesias M., Sevillano M., Palomo-Ponce S., Tauriello D., Byrom D. (2015). Stromal gene expression defines poor-prognosis subtypes in colorectal cancer. Nat. Genet..

[2] Wu F., Wang J., Li M., Wang H. (2018). Biomolecular networks for complex diseases. Complexity.

[3] Lu C., Yang M., Luo F., Wu F., Li M., Pan Y., Li Y., Wang J. (2018). Prediction of lncRNA-disease associations based on inductive matrix completion. Bioinformatics.

[4] Consortium T.G. (2015). The Genotype-Tissue Expression (GTEx) pilot analysis: Multitissue gene regulation in humans. Science.

[5] Wu L., Li M., Wang J., Wu F. (2018). CytoCtrlAnalyser: A Cytoscape app for biomolecular network controllability analysis. Bioinformatics.

[6] Li H.D., Bai T., Sandford E., Burmeister M., Guan Y. (2018). BaiHui: Cross-species brain-specific network built with hundreds of hand-curated datasets. Bioinformatics.

[7] Buenrostro J.D., Wu B., Litzenburger U.M., Ruff D., Gonzales M.L., Snyder M.P., Chang H.Y., Greenleaf W.J. (2015). Single-cell chromatin accessibility reveals principles of regulatory variation. Nature.

[8] Trapnell C., Cacchiarelli D., Grimsby J., Pokharel P., Li S., Morse M., Lennon N., Livak K., Mikkelsen T., Rinn J. (2014). The dynamics and regulators of cell fate decisions are revealed by pseudo temporal ordering of single cells. Nat. Biotechnol..

[9] Diaz A., Liu S., Sandoval C., Pollen A., Nowakowski T., Lim D., Kriegstein A. (2016). SCell: Integrated analysis of single-cell RNA-seq data. Bioinformatics.

[10] Jia C., Hu Y., Kelly D., Kim J., Li M., Zhang N. (2017). Accounting for technical noise in differential expression analysis of single-cell RNA sequencing data. Nucleic Acids Res..

[11] Li J., Klughammer J., Farlik M., Penz T., Spittler A., Barbieux C., Berishvili E., Bock C., Kubicek S. (2016). Single-cell transcriptomes reveal characteristic features of human pancreatic islet cell types. EMBO Rep..

[12] Buganim Y., Faddah D.A., Cheng A.W., Itskovich E., Markoulaki S., Ganz K., Klemm S.L., Oudenaarden A., Jaenisch R. (2012). Single-cell expression analyses during cellular reprogramming reveal an early stochastic and a late hierarchic phase. Cell.

[13] Wang Y., Li M., Zheng R., Shi X., Li Y., Wu F., Wang J. (2018). Using Deep Neural Network to Predict Drug Sensitivity of Cancer Cell Lines. Intelligent Computing Theories and Application.

[14] Stegle O., Teichmann S.A., Marioni J.C. (2015). Computational and analytical challenges in single-cell transcriptomics. Nat. Rev. Genet..

[15] Pouyan M.B., Kostka D. (2018). Random forest-based similarity learning for single cell RNA sequencing data. Bioinformatics.

[16] Grün D., Lyubimova A., Kester L., Wiebrands K., Basak O., Sasaki N., Clevers H., Oudenaarden A. (2015). Single-cell messenger RNA sequencing reveals rare intestinal cell types. Nature.

[17] Shalek A.K., Satija R., Adiconis X., Gertner R.S., Gaublomme J.T., Raychowdhury R., Schwartz S., Yosef N., Malboeuf C., Lu D. (2013). Single-cell transcriptomics reveals bimodality in expression and splicing in immune cells. Nature.

[18] Wen Y., Wei Y., Zhang S., Li S., Liu H., Wang F., Zhao Y., Zhang D., Zhang Y. (2016). Cell subpopulation deconvolution reveals breast cancer heterogeneity based on DNA methylation signature. Brief. Bioinform..

[19] Chen H., Guo J., Mishra S.K., Robson P., Niranjan M., Zheng J. (2015). Single-cell transcriptional analysis to uncover regulatory circuits driving cell fate decisions in early mouse development. Bioinformatics.

[20] Peng X., Wang J., Peng W., Wu F., Pan Y. (2017). Protein-protein interactions—Detection, reliability assessment and applications. Brief. Bioinform..

[21] Wang H., Wang J., Zhou L. (2018). A survival ensemble of extreme learning machine. Appl. Artif. Intell..

[22] Zeng M., Li M., Fei Z., Yu Y., Pan Y., Wang J. (2019). Automatic ICD-9 coding via deep transfer learning. Neurocomputing.

[23] Liu J., Li M., Lan W., Wu F.X., Pan Y., Wang J. (2018). Classification of Alzheimer’s disease using whole brain hierarchical network. IEEE/ACM Trans. Comput. Biol. Bioinform..

[24] Shekhar K., Lapan S.W., Whitney I.E., Tran N.M., Macosko E.Z., Kowalczyk M., Adiconis X., Levin J.Z., Nemesh J., Goldman M. (2016). Comprehensive classification of retinal bipolar neurons by single-cell transcriptomics. Cell.

[25] Chen J., Shao J. (2000). Nearest neighbor imputation for survey data. J. Off. Stat..

[26] Wu X., Kumar V., Quinlan R.R., Ghosh J., Yang Q., Motoda H., McLachlan G.J., Ng A., Liu B., Yu P.S. (2008). Top 10 algorithms in data mining. Knowl. Inf. Syst..

[27] Fortunato S. (2009). Community detection in graphs. Phys. Rep..

[28] Rubinov M., Sporns O. (2010). Complex network measures of brain connectivity: Uses and interpretations. NeuroImage.

[29] Newman M.E., Girvan M. (2004). Finding and evaluating community structure in networks. Phys. Rev. E.

[30] Llorens-Bobadilla E., Zhao S., Baser A., Saiz-Castro G., Zwadlo K., Martin-Villalba A. (2015). Single-cell transcriptomics reveals a population of dormant neural stem cells that become activated brain injury. Cell Stem Cell.

[31] Darmanis S., Sloan S.A., Zhang Y., Enge M., Caneda C., Shuer L.M., Gephart M.G., Barres B.A., Stephen R., Quake S.R. (2015). A survey of human brain transcriptome diversity at the single cell level. Proc. Natl. Acad. Sci. USA.

[32] Corpet F. (1988). Multiple sequence alignment with hierarchical clustering. Nucleic Acids Res..

[33] Shin J., Berg D.A., Zhu Y., Shin J.Y., Song J., Bonaguidi M.A., Enikolopov G., Nauen D.W., Christian K.M., Ming G. (2015). Single-cell RNA-seq with waterfall reveals molecular cascades underlying adult neurogenesis. Cell Stem Cell.

[34] Dhillon I.S., Guan Y., Kulis B. Kernel k-means: Spectral clustering and normalized cuts. Proceedings of the 10th ACM SIGKDD International Conference on Knowledge Discovery and Data Mining.

[35] Jin R., Goswami A., Agrawal G. (2006). Fast and exact out-of-core and distributed k-means clustering. Knowl. Inf. Syst..

[36] Xu C., Su Z. (2015). Identification of cell types from single-cell transcriptomes using a novel clustering method. Bioinformatics.

[37] Shao C., Höfer T. (2016). Robust classification of single-cell transcriptome data by nonnegative matrix factorization. Bioinformatics.

[38] Kiselev V.Y., Kirschner K., Schaub M.T., Andrews T., Yiu A., Chandra T., Natarajan K.N., Reik W., Barahona M., Green A.R. (2017). SC3: Consensus clustering of single-cell RNA-seq data. Nat. Methods.

[39] Wang B., Zhu J., Pierson E., Ramazzotti D., Batzoglou S. (2017). Visualization and analysis of single-cell RNA-seq data by kernel-based similarity learning. Nat. Methods.

[40] Liu J., Wang X., Zhang X., Pan Y., Wang X., Wang J. (2018). MMM—Classification of schizophrenia using multi-modality multi-atlas feature representation and multi-kernel learning. Multimed. Tools Appl..

[41] Zhang S. (2012). Nearest neighbor selection for iteratively kNN imputation. J. Syst. Softw..

[42] Zhu X., Qiu J., Xie M., Wang J. (2017). A multi-objective biclustering algorithm based on fuzzy mathematics. Neurocomputing.

[43] Zhang S., Li X., Zong M., Zhu X., Cheng D. (2017). Learning *k* for kNN Classification. ACM Trans. Intell. Syst. Technol..

[44] Zhang S., Li X., Zong M., Zhu X., Wang R. (2017). Efficient kNN classification with different numbers of nearest neighbors. IEEE Trans. Neural Netw. Learn. Syst..

[45] Luo H., Li M., Wang S., Liu Q., Li Y., Wang J. (2018). Computational drug repositioning using low-rank matrix approximation and randomized algorithms. Bioinformatics.

[46] Li M., Yang J., Wu F., Pan Y., Wang J. (2018). DyNetViewer—A Cytoscape app for dynamic network construction, analysis and visualization. Bioinformatics.

[47] Li A., Li J., Pan Y., Yin X., Yong X. (2015). Homophyly/Kinship model: Naturally evolving networks. Sci. Rep..

[48] Kong Y., Gao J., Xu Y., Pan Y., Wang J., Liu J. (2019). Classification of autism spectrum disorder by combining brain connectivity and deep neural network classifier. Neurocomputing.

[49] Aibar S., González-Blas C.B., Moerman T., Huynh-Thu V.A., Imrichova H., Hulselmans G., Rambow F., Marine J., Geurts P., Aerts J. (2017). SCENIC: Single-cell regulatory network inference and clustering. Nat. Methods.

[50] Nowicka M., Krieg C., Weber L.M., Hartmann F.J., Guglietta S., Becher B., Levesque M.P., Robinson M.D. (2017). CyTOF workflow: Differential discovery in high-throughput high-dimensional cytometry datasets. F1000Research.

[51] Lin C., Jain S., Kim H., Bar-Joseph Z. (2017). Using neural networks to improve single-cell RNA-seq data analysis. bioRxiv.

[52] Cover T., Hart P. (1967). Nearest neighbor pattern classification. IEEE Trans. Inf. Theory.

[53] Li A., Yin X., Pan Y. (2016). Three-dimensional gene map of cancer cell types: Structural entropy minimisation principle for defining tumour subtypes. Sci. Rep..

[54] Bacher R., Kendziorski C. (2016). Design and computational analysis of single-cell RNA-sequencing experiments. Genome Biol..

[55] Grün D., Lennart Kester L., Oudenaarden A. (2014). Validation of noise models for single-cell transcriptomics. Nat. Methods.

[56] Biase F., Cao X., Zhong S. (2014). Cell fate inclination within 2-cell and 4-cell mouse embryos revealed by single-cell RNA sequencing. Genome Res..

[57] Yan L., Yang M., Guo H., Yang L., Wu J., Li R., Liu P., Lian Y., Zheng X., Yan J. (2013). Single-cell RNA-seq profiling of human preimplantation embryos and embryonic stem cells. Nat. Struct. Mol. Biol..

[58] Deng Q., Ramsköld D., Reinius B., Sandberg R. (2014). Single-cell RNA-seq reveals dynamic, random monoallelic gene expression in mammalian cells. Science.

[59] Pollen A., Nowakowski T., Shuga J., Wang X., Leyrat A., Lui J., Li N., Szpankowski L., Fowler B., Chen P. (2014). Low-coverage single-cell mRNA sequencing reveals cellular heterogeneity and activated signaling pathways in developing cerebral cortex. Nat. Biotechnol..

[60] Treutlein B., Brownfield D.G., Wu A.R., Neff N.F., Mantalas G.L., Espinoza F.H., Desai T.J., Krasnow M.A., Quake S.R. (2014). Reconstructing lineage hierarchies of the distal lung epithelium using single-cell RNA-seq. Nature.

[61] Patel A.P., Tirosh I., Trombetta J.J., Shalek A.K., Gillespie S.M., Wakimoto H., Cahill D.P., Nahed B.V., Curry W.T., Martuza R.L. (2014). Single-cell RNA-seq highlights intratumoral heterogeneity in primary glioblastoma. Science.

[62] Chung W., Eum H.H., Lee H., Lee K., Lee H., Kim K., Ryu H.S., Kim S., Lee J.E., Park Y.H. (2017). Single-cell RNA-seq enables comprehensive tumour and immune cell profiling in primary breast cancer. Nat. Commun..

[63] Ramsköld D., Luo S., Wang Y., Li R., Deng Q., Faridani O.R., Daniels G.A., Khrebtukova I., Loring J.F., Laurent L.C. (2012). Full-length mRNA-Seq from single-cell levels of RNA and individual circulating tumor cells. Nat. Biotechnol..

[64] Hunter J.D. (2007). Matplotlib: A 2D graphics environment. Comput. Sci. Eng..

[65] Zhu X., Li X., Zhang S. (2016). Block-row sparse multiview multilabel learning for image classification. IEEE Trans. Cybern..

