# Supplementary material for: A Hybrid Clustering Algorithm for Identifying Cell Types from Single-Cell RNA-Seq Data
Source: Genes (Basel). 2019 Jan 29;10(2):98. doi: 10.3390/genes10020098 (PMC6409843; doi:10.3390/genes10020098)
Supplement: Supplementary file 1 [file genes-10-00098-s001.pdf]

Supplementary file for

A Hybrid Clustering Algorithm for Identifying Cell Types from Single-cell RNA-seq Data

Xiaoshu Zhu[1,2], Hong-Dong Li[1], Yunpei Xu[1], Lilu Guo[2], Fang-Xiang Wu[3] , Guihua Duan[1,*] and Jianxin Wang

[1] School of Information Science and Engineering, Central South University, Changsha, Hunan, China; xszhu@csu.edu.cn; hongdong@csu.edu.cn; xu_yunpei@csu.edu.cn; jxwang@mail.csu.edu.cn

[2] School of Computer Science and Engineering, Yulin Normal University, Yulin, Guangxi, China; jgxygll@163.com

[3] Division of Biomedical Engineering and Department of Mechanical Engineering, University of Saskatchewan, Saskatoon, SKS7N5A9, Canada; faw341@mail.usask.ca

List of supplementary content:

**Supplementary Figure S1**	The scatter diagram of eight datasets by t-SNE.
**Supplementary Figure S2**	The three-dimensional scatter diagram of eight datasets by PCA.
**Supplementary Table S1**	Cluster results comparison between SSE and NMF, SIMLR, SE in terms of NMI
**Supplementary Table S2**	Cluster results comparison between SSE and NMF, SIMLR, SE in terms of ARI

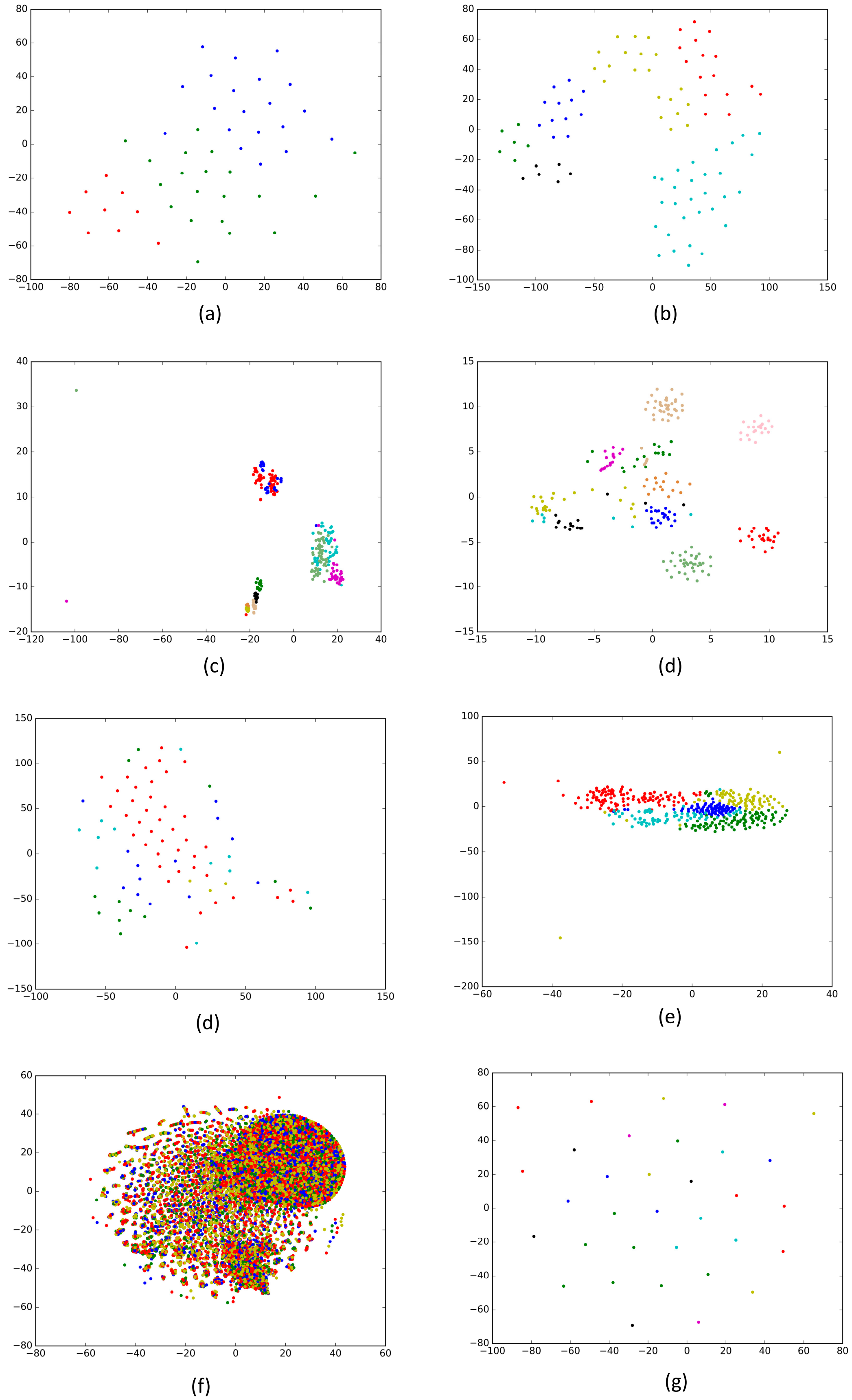

Figure S1. The scatter diagram of eight datasets by t-SNE. (a) Biase; (b) Yan; (c) Deng; (d) Pollen; (e) Treutlein; (f) Patel; (g) Chung; (h) Ramskold.

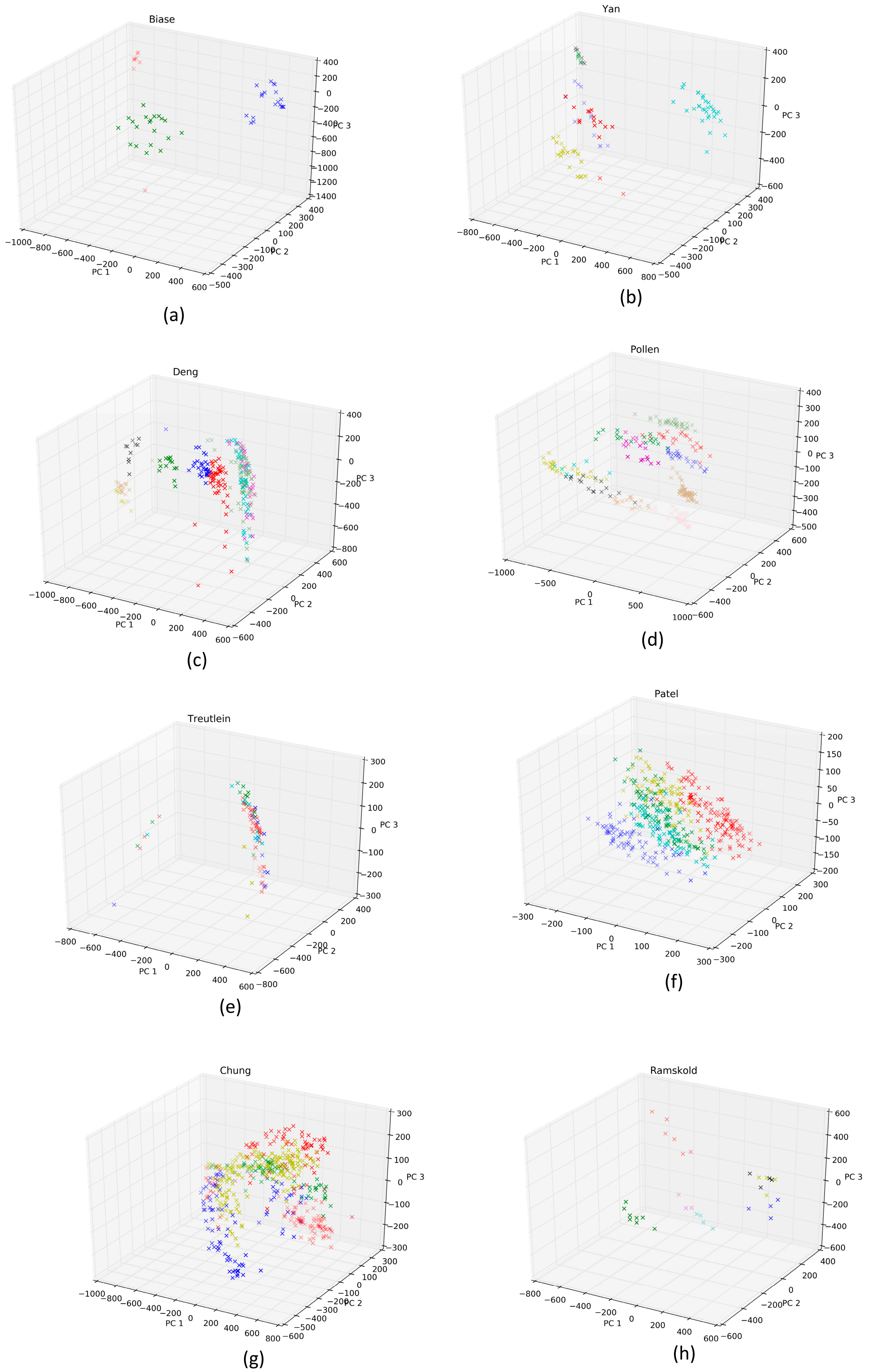

Figure S2. The three-dimensional scatter diagram of eight datasets by PCA. (a) Biase; (b) Yan; (c) Deng; (d) Pollen; (e) Treutlein; (f) Patel; (g) Chung; (h) Ramskold.

Table S1. Cluster result comparison between SSE and NMF, SIMLR, SE in terms of NMI

GSE/ID	Datasets	NMF	SIMLR	SE
GSE57249	Biase	0.358	0.920	0.718
GSE36552	Yan	0.745	0.941	0.841
GSE45719	Deng	0.547	0.917	0.837
E-MTAB-2805	Pollen	0.962	1.000	0.794
GSE52583	Treutlein	0.508	0.909	0.334
GSE57872	Patel	0.531	0.753	NA
GSE75688	Chung	0.331	0.411	0.416
GSE38495	Ramskold	0.736	0.922	0.597

Table S2. Cluster result comparison between SSE and NMF, SIMLR, SE in terms of ARI

GSE/ID	Datasets	NMF	SIMLR	SE
GSE57249	Biase	0.246	0.927	0.651
GSE36552	Yan	0.647	0.826	0.670
GSE45719	Deng	0.299	0.730	0.682
E-MTAB-2805	Pollen	0.936	1.000	0.630
GSE52583	Treutlein	0.242	0.744	0.208
GSE57872	Patel	0.434	0.557	NA
GSE75688	Chung	0.118	0.335	0.239
GSE38495	Ramskold	0.559	0.844	0.468
